# Experimental Tests for Heritable Morphological Color Plasticity in Non-Native Brown Trout (*Salmo trutta*) Populations

**DOI:** 10.1371/journal.pone.0080401

**Published:** 2013-11-18

**Authors:** Peter A. H. Westley, Ryan Stanley, Ian A. Fleming

**Affiliations:** Department of Ocean Sciences and Department of Biology, Memorial University, St. John’s, Newfoundland, Canada; University of California, Berkeley, United States of America

## Abstract

The success of invasive species is frequently attributed to phenotypic plasticity, which facilitates persistence in novel environments. Here we report on experimental tests to determine whether the intensity of cryptic coloration patterns in a global invader (brown trout, *Salmo trutta*) was primarily the result of plasticity or heritable variation. Juvenile F_1_ offspring were created through experimental crosses of wild-caught parents and reared for 30 days in the laboratory in a split-brood design on either light or dark-colored gravel substrate. Skin and fin coloration quantified with digital photography and image analysis indicated strong plastic effects in response to substrate color; individuals reared on dark substrate had both darker melanin-based skin color and carotenoid-based fin colors than other members of their population reared on light substrate. Slopes of skin and fin color reaction norms were parallel between environments, which is not consistent with heritable population-level plasticity to substrate color. Similarly, we observed weak differences in population-level color within an environment, again suggesting little genetic control on the intensity of skin and fin colors. Taken as whole, our results are consistent with the hypothesis that phenotypic plasticity may have facilitated the success of brown trout invasions and suggests that plasticity is the most likely explanation for the variation in color intensity observed among these populations in nature.

## Introduction

In the broadest sense, phenotypic plasticity is the ability of an individual to respond to an environmental stimulus with a change in behavioral state, morphological form, or physiological functioning [1]. Adaptive phenotypic plasticity (i.e., plasticity that increases fitness) can facilitate the colonization of new habitats [2], [3], allow populations to track climate change [4], and reduce the probability of predation through inducible defenses [5]. In addition, phenotypic plasticity is frequently implicated in the successful establishment and spread of non-native invasive species [6], [7], though consensus on its importance to invasion has not been reached [8]. On-going debate notwithstanding, empirical evidence suggests that plasticity likely plays an important role in the successful establishment of at least certain groups of organisms such as freshwater fishes [9]–[11]. For example, phenotypic plasticity in the intensity of coloration has recently been proposed as a mechanism for the successful colonization by coastrange sculpin (*Cottus aleuticus*) of newly formed freshwater environments in Alaska [12]–[14].

The intensity of fish coloration is often assumed to be largely the result of phenotypic plasticity. This likely stems, at least in part, from the observation that carotenoid pigments responsible for yellow and red colors are primarily dependent on uptake from the environment [15], though some species of fish (e.g., guppies, *Poecilia reticulata*) can supplement carotenoid colors through self-synthesis [16], [17]. Carotenoid-based colors are used for reproductive display in guppies [18], sticklebacks (*Gasterosteus aculeatus*) [19] and sockeye salmon (*Oncorhynchus nerka*) [20], and frequently tested as signals for ‘good genes’ [21]. Whereas careotenoid-based colors are at least in part dependent on uptake from the environment, melanin-based colors (browns and blacks) can be synthesized directly in the specialized pigment organelles, melanosomes, of individuals [22]. In salmonid fishes, the melanin-based colors are thought to be involved primarily in cryptic camouflage [23], [24], though they may also have a role in spawning displays and apparently can influence reproductive success [21]. Generally speaking, plasticity in melanin-based cryptic coloration in freshwater fishes is under both neural and hormonal regulation [22]. Nearly instantaneous change in color is termed ‘physiological color change’ and results from neural control of pigment cell aggregation of chromatosomes into the perikaryon or dispersion throughout the cytoplasm. In contrast, ‘morphological color change’ occurs over weeks or months and results from hormonal regulation of α-melanophore-stimulating hormone (α-MSH) and associated synthesis or decay of chromatophores themselves [22]. Consistent with the observation that morphological color change allows individuals to match their surroundings, empirical studies have revealed reduced predation on individuals that were acclimated to substrate colors similar to conditions they would later experience in the wild [13], [25].

Plasticity often has a heritable basis, where the relationship between the environment and expressed phenotype is termed the norm of reaction, or reaction norm [26]. To the extent that the reaction norm has a genetic underpinning, selection acting on plasticity can lead to an evolutionary response [27], [28]. Thus, the genetic basis of color plasticity may have implications for the evolutionary trajectories of populations colonizing new environments [29]. Recent work has revealed family-level differences in color plasticity to rearing substrate in a putative ancestral source of newly formed coastrange sculpin populations [14]. This finding suggests that heritable responses to rearing environments consistent with genotype by environment interactions may lead to the evolution of population-specific color reaction norms in young fish populations.

The salmonidae family, of which brown trout is a member, is renowned for remarkable variation in life history, behavior, and morphology [30]–[32]. Depending on the trait, phenotypic variation in salmonids can primarily be attributed to plasticity [33], [34], [35] or heritable genetic variation [36]–[39]. Brown trout populations vary greatly in morphology [40], including in melanin-based [21] and carotenoid-based pigmentation patterns [41], [42]. Coloration patterns often vary sufficiently to distinguish among populations in nature [43]. The repeated global introductions of brown trout are frequently assumed to have been successful by the appreciable plasticity demonstrated in the species [40], [44], [45]. However, plasticity and local adaptation do not have to be mutually exclusive. Indeed, recent evidence from our research group has demonstrated the contemporary evolution of local adaptation by non-native populations of brown trout in Newfoundland, Canada [46]. This suggests that plasticity alone may not entirely underpin the ability of brown trout to adaptively respond to new environmental conditions.

Here we quantify the plasticity and population-specific norms of reaction in cryptic coloration of locally adapted non-native populations of brown trout (*Salmo trutta*) [47]. Wild-caught individuals from the populations of brown trout examined in this study differ in a suite of phenotypic traits, including skin color intensity (i.e., lightness vs. darkness of coloration), and differences are correlated with habitat features [48]. Specifically, dark colorations tend to correlate with dark environments and vice versa. Given the observed local adaptation and correlation between cryptic coloration and environmental features, we tested the hypothesis that differences in coloration patterns would be maintained in a common environment consistent with heritable trait divergence. Additionally we hypothesized that populations from relatively homogeneous environments would display less plasticity in skin coloration than populations from relatively heterogeneous environments. Our experiment addressed the following specific questions: 1) is skin coloration plastic between rearing environments? (i.e., the slope of the reaction norms ≠ zero), 2) do populations differ in coloration not due to plasticity? (i.e., zero slope of reaction norms but different y-intercepts), 3) do populations exhibit plasticity and differences in coloration? (i.e., non-zero parallel slopes of reaction norm and different y-intercepts), and 4) are population-level responses consistent with genotype by environment interactions? (i.e., non-parallel slopes of reaction norms).

## Methods

To test these hypotheses, we employed a replicated-randomized laboratory experiment using juvenile lab-born F_1_ offspring of wild-caught parents. We created 28 full-sib families by crossing unique sires and dams caught from Middle Rocky Brook (n = 8), Parkers Pond Brook (n = 5), Rennies River (n = 8), and Waterford River (n = 7), Newfoundland, Canada. More extensive details on the capture, crosses, and habitats inhabited by the Middle Rocky, Rennies, and Waterford populations are available in previous papers [46], [48], [49]. Briefly, the Middle Rocky and Parkers Pond Brook populations inhabit very similar, relatively homogenous environments; both are short (ca. 2.5 km), high gradient streams (> 6% ) with visually dark substrate and extensive canopy cover which limits the amount of light reaching the streams. In contrast, the Rennies and Waterford habitats are larger (> 10 km), lower gradient (< 2%), have less canopy cover and lighter substrate color, and are more heterogonous in these features. Patterns of potential gene flow also differ among populations. Gene flow is only possible between population pairs: Middle Rocky and Parkers Pond Brook populations are isolated from the other populations but separated from each other by only a few 100 meters. Thus based on these combinations of habitat similarity and potential for genetic exchange, we predicted that the population pairs of Middle Rocky and Parkers Pond Brook, and the Rennies and the Waterford would be more similar to each other than to the other populations.

Families were incubated separately through the larval alevin stage in Heath trays, but upon successful transition to exogenous feeding (i.e., emergence) were mixed into communal white colored population-specific holding tanks as space limitations precluded family-level rearing. Lights were maintained on a cycle to emulate the ambient photoperiod. Fish were fed a combination of *Artemia* nauplii and commercial aquaculture food *ad libitum* four-eight times daily. Approximately a month after the final family initiated feeding (timing spanned two weeks resulting from different spawn timing), we initiated a split-brood experimental design where individuals from each population were randomly assigned to two treatments: 1) white-sided artificial streams with white-colored marble gravel (hereafter *Light* substrate) or 2) white-sided artificial streams with dark-grey crushed gravel (hereafter *Dark* substrate). Thirty individuals from each of the four populations were added to each treatment and reared in isolation from the other populations. Each treatment was then replicated four times. This design thus yielded a grand N = 32 from n = 4 *Light* and n = 4 *Dark* for each of four populations. Individuals were reared for 30 days in experimental streams, maintained at ambient water temperatures (mean  =  12°C), lighting maintained at a 12:12 hr cycle, and fed dripped *Artemia* nauplii from feeders twice per day. *Artemia* were used in lieu of commercial fish feed as they are rich in carotenoid pigments [50] and because excess dry feed is extremely difficult to clean from these experimental streams. The 30 day length of the experiment was determined based on the length of time reportedly necessary to ensure morphological color change [22]. Additional details on the experimental streams can be found in Oke et al. [51]. Mortalities (n  =  211 of 960, spread randomly among treatments and populations) were removed daily and live replacements from the same population were added to the streams to maintain rearing densities; however, replacement fish (denoted by clipped adipose fin) were excluded from analyses.

### Morphological color quantification

**Figure 1:**
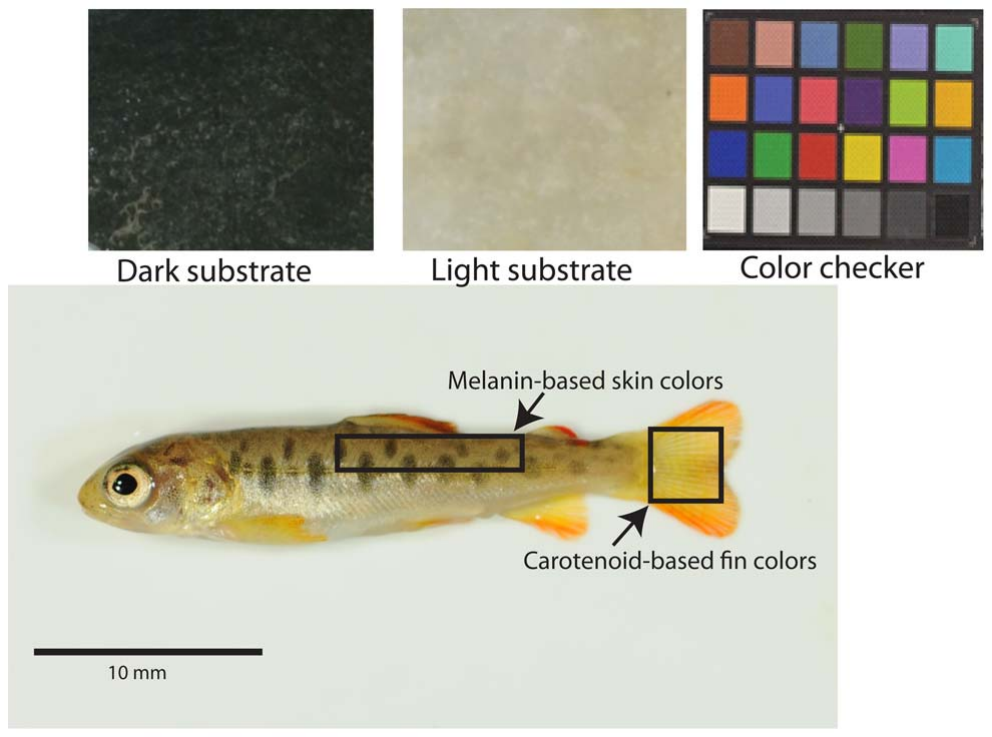
Example photograph of brown trout showing regions used in color analyses. A 32(top). Areas cropped for analyses of melanin and carotenoid-based colors are shown along with the X-rite color checker card.

To ensure that our measurements reflected morphological rather than physiological color change, we allowed individuals to acclimate to white-sided containers for at least 10 minutes prior to photographing [13]. Following acclimation, individuals were lightly anaesthetized with tricaine methanesulfonate (MS-222), weighed to the nearest 0.0001 g on an analytical balance, and photographed with a Nikon D300 and 60 mm Micro Nikkor lens using manual white balance settings and low compression JPEG format under four ‘natural daylight’ compact florescent bulbs. Photographs were taken in a standardized position and each image included a Munsell X-rite color checker card (X-rite, Inc., Grand Rapids, MI, USA), which was subsequently used to correct for subtle differences in lighting or exposure (Fig. 1). Spreading of the caudal fin was standardized to the best of our ability, but variation in fin size and shape varied among individuals. Regardless, we handled all populations and treatments similarly to minimize any potential bias resulting from the photography process. The same procedure was used to photograph individuals at the start and end of the 30 day experimental period. After necessary data were collected from lightly anesthetized individuals after the 30 day experimental period, fish were then euthanized in an overdose of MS-222.

Images were prepared for analyses in Adobe Photoshop CS3 ® (Adobe Systems Incorporated, San Francisco, CA, USA) prior to quantifying color. Specifically, we cropped each standardized photograph from two areas on the fish, denoted by homologous landmarks (Fig. 1), to assess melanin- (cropped dorsal and lateral areas) and carotenoid-based colors (cropped caudal fins). We opted to analyze two separate regions as the colors are differentially expressed in different areas of the body [42] and because we wanted independent measures of color for analyses.

The white vignette of the Munsell card was cropped and digitized as a three dimensional red-green-blue (RGB) color array using the Matlab 2012a image analysis toolbox. For each cropped vignette, color calibration coefficients were calculated as the percent difference between the average value of each RGB spectra and the corresponding Munsell set points for the white vignette (RGB: 243,243,242). Calibration coefficients were then used to calibrate images to a common standard [52]. We calibrated images in groups of 30 individuals, corresponding to given populations and experimental treatments.

Following calibration, images were processed for glare, defined as white saturated pixels (i.e., when RGB values all exceeded 240). Pixels identified as glare were assigned RGB values derived as a weighted mean of all surrounding non-glare pixels. This provides a method by which to remove potential biases imparted by image specific glare features. Lastly, image quantization was employed in MATLAB to smooth image color, reducing each image to 20 base Red-Green-Blue (RGB) groupings of similar color for statistical summary.

Principal components analysis was then used to reduce the 20 RGB pallet data to two dimensions. For each image, a weighted average PCA score was calculated according to the following equation:
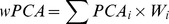
where *PCA_i_* is the PCA score for the 20 RGB color pallets of percent coverage *W_i_*, where the weight was defined as the number of pixels per color pallet divided by the total number of pixels in the image. The resulting first wPCA is highly correlated (Pearson r  =  –0.99) with L* values of the more common International Commission on Illumination (CIE) 1976 L*a*b* color space models, used recently by [12]–[14] as a measure of color lightness or darkness. CIE color space model provides three indices of color summary: the lightness axis (L*) where higher values indicate lighter color, the red-green axis (a*) where higher values indicate redder colors, and the yellow-blue axis (b*) where higher values indicate yellow colors [53]. The primary benefit of using the wPCA approach was that it allowed a direct interpretation of color by maintaining size-adjusted RGB color values for each individual (see Supplemental Figs. S1–S2).

### Data analysis

**Figure 2:**
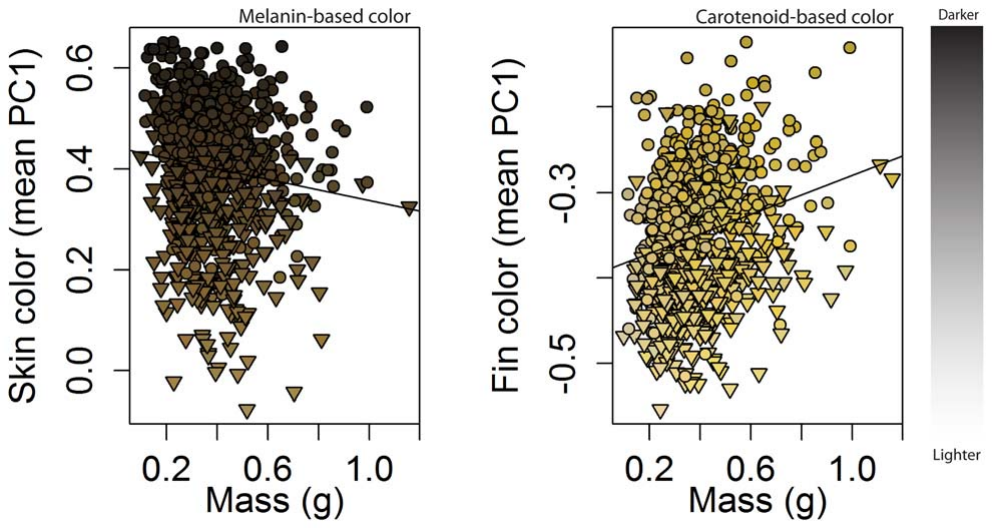
Relationship between body mass (g) and melanin-based and carotenoid-based skin color (weighted mean PC1) of brown trout reared for 30 days on dark substrate (circles) or light substrate (triangles). Each point is colored by its corresponding wPC1 RGB color combination to facilitate interpretation of color differences among individuals and treatments. Lines are the best-fit regressions used to correct for size. Note different y-axes.

To control for allometric size effects, we used residuals from a fitted ordinary least squares relationship between body size (in mass) and melanin-based skin color and carotenoid-based fin color. Melanin-based skin colors were lighter with mass (OLS, slope = –0.11, p<0.001, r^2^ = 0.02, Fig. 2a) while carotenoid-based fin colors yielded the opposite relationship (OLS, slope  = 0.10, p<0.001, r^2^ = 0.05, Fig. 2b). Residuals were normally-distributed and subsequent analyses met parametric assumptions. As we were unable to track the plasticity at the individual-level, we used the average color of individuals from each experimental replicate as our unit of replication (grand N = 32). Size-corrected skin and skin color values were then used in fixed-effect general linear models (GLM) formulated to test the following *a priori* hypotheses and assessed in a selection framework based on AICc [54]:

1. Populations are plastic (GLM, non-zero slopes): 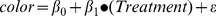

2. Populations differ in color but not plasticity (GLM, different intercepts) 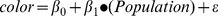

3. Populations are both plastic and differ in color (GLM, non-zero slopes, intercepts differ): 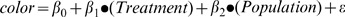

4. Populations differ in shape of reaction norms: 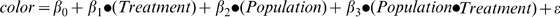

We interpreted the interaction term in model (4) as evidence of genotype by environment interactions and considered an interaction to be consistent with heritable differences in plastic response [14], [26]. To test whether populations differed in the magnitude of their plastic response within an environment, we also used the difference in the average color between the beginning and end of the experiment (final-initial average color). All statistical analyses were done in R 2.15.2 [55].

### Ethics statement

All necessary permits were obtained to capture and transport live specimens from the Department of Fisheries and Ocean Sciences, St. John’s, Newfoundland. Handling and housing of the experimental animals were done in accordance with the guidelines provided by the Canadian Council on Animal Care and with approval of Memorial University’s Institutional Animal Care Committee (09-10-IF).

## Results

**Figure 3:**
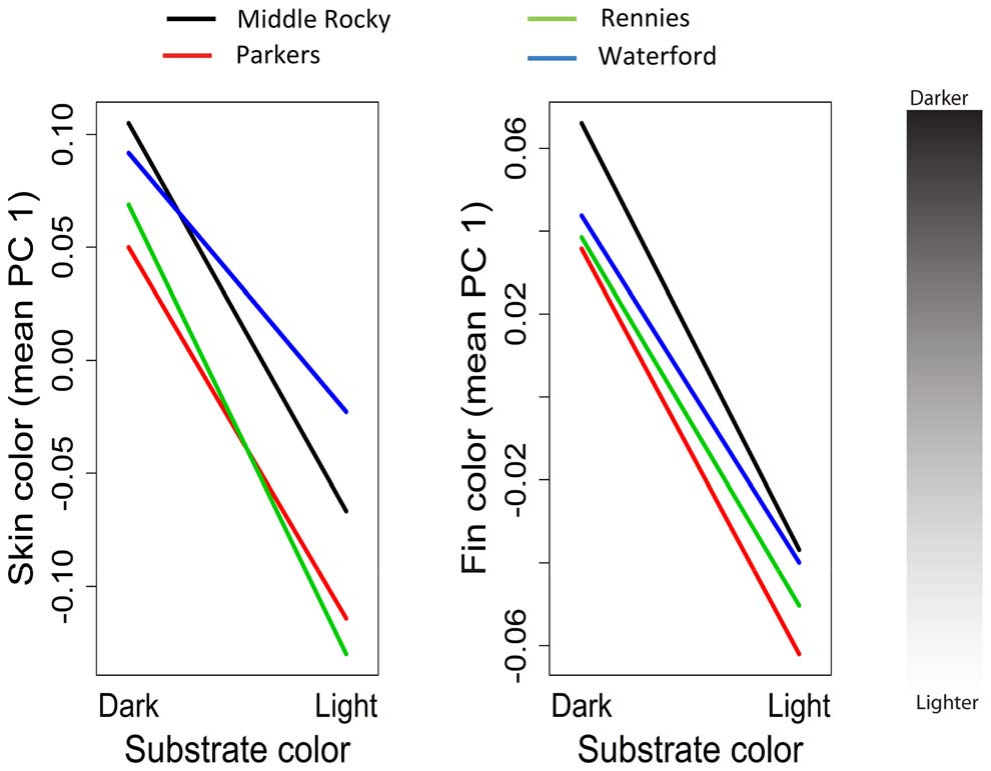
Norms of reaction among four populations of introduced brown trout in a) melanin-based and b) carotenoid-based skin coloration (size-corrected) as a function of substrate color. Note different y-axes. Means and associate error are omitted to facilitate visualization of the plastic response to substrate color.

General linear modeling and model selection revealed *i*) that skin and fin color was highly plastic (non-zero slopes of reaction norms), *ii*) little evidence of population differences in color within environments (y-intercepts did not differ), *iii*) little evidence of population differences in extent of plasticity, and *iv*) no evidence of population-specific shape of reaction norms (Fig. 3, Table 1). Evidence for differences among populations were only apparent in comparisons of melanin-based skin color after 30 days of rearing (Table 1), yet the model that included only a treatment effect (e.g., light vs. dark substrate) also received substantial support.

**Table 1:** Model selection results for tests of heritable color plasticity in non-native Newfoundland brown trout.

Response	Model	k	ΔAIC_c_	AIC_c_ Weight	Cumulative Weight	r^2^
Final melanin-based color	**Treatment (E) + Population (G)**	6	0.00	0.66	0.66	0.76
	Treatment (E)	3	1.42	0.32	0.98	0.67
	Treatment (E) x Population (G)	9	7.56	0.02	1.00	0.78
	Null	1	31.90	0.00	1.00	NA
	Population (G)	5	39.10	0.00	1.00	0.09
Final carotenoid-based color	**Treatment (E)**	3	0.00	0.94	0.94	0.64
	Treatment (E) + Population (G)	6	5.65	0.06	1.00	0.67
	Treatment (E) x Population (G)	9	16.07	0.00	1.00	0.67
	Null	1	27.61	0.00	1.00	NA
	Population (G)	5	36.77	0.00	1.00	0.03
Plasticity melanin-based color	**Treatment (E)**	3	0.00	0.93	0.93	0.52
(final-initial color)	Treatment (E) + Population (G)	6	5.40	0.06	0.99	0.56
	Treatment (E) x Population (G)	9	14.90	0.00	0.99	0.58
	Null	1	18.60	0.00	1.00	NA
	Population (G)	5	27.30	0.00	1.00	0.04
Plasticity carotenoid-based color	**Treatment (E)**	3	0.00	0.55	0.55	0.53
(final-initial color)	**Treatment (E) + Population (G)**	6	0.42	0.44	0.99	0.64
	Treatment (E) x Population (G)	9	10.33	0.00	1.00	0.65
	Null	1	19.50	0.00	1.00	NA
	Population (G)	5	26.10	0.00	1.00	0.10

K is the number of parameters in the models, and AIC_c_ is the small sample size corrected Akaike Information Criterion. Models with ΔAICc scores of < 2 are considered plausible and denoted in bold. Treatment is the effect of substrate color (a proxy for environmental effects, E), Population is a proxy for genetic effects (G).

**Figure 4:**
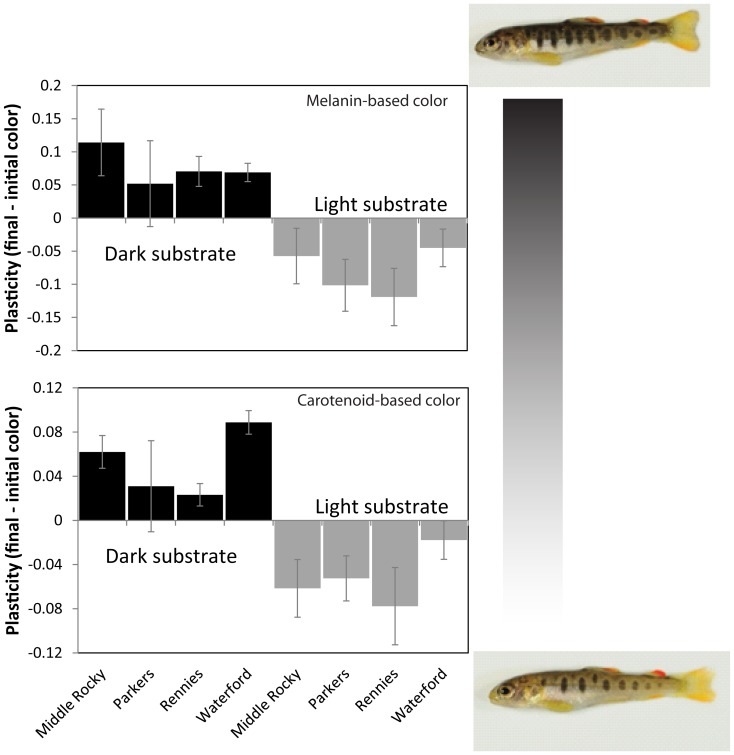
Color plasticity in four brown trout populations. Average (±1 SE) plasticity (final-initial skin color) among four brown trout populations after 30 days of rearing on either dark or light substrate. Photographs of representative individuals from the Middle Rocky population (one reared on light substrate the other on dark) are shown to visualize color extremes.

Similarly, the extent of color plasticity inferred from change in color between the end and beginning of the experiment differed primarily as a function of the environment (Fig. 4, Table 1). Again, individuals reared on dark substrate exhibited darker melanin and carotenoid-based colors than other members of their population reared on light substrate. Population differences in carotenoid based-colors were detected (Table 1), but the model with only an environmental effect received greater support.

Counter to predictions based on habitat similarity and potential gene flow, the Middle Rocky and Parkers Pond Brook did not exhibit more similar color intensity or plastic responses than the other populations, which in turn were predicted to be more similar to each other. Indeed, Fig. 3. suggests precisely the opposite, that Middle Rocky and the Waterford populations are more similar to each other than they are to the Rennies and Parkers Pond Brook.

## Discussion

Here we report on an experimental test for heritable differences in the plasticity of skin and fin color intensity to rearing substrate among populations of non-native brown trout, established in new environments for approximately 130 years. Our primary finding was that populations displayed marked color plasticity in response to their rearing environment, but the shapes of the responses (i.e., the reaction norms) did not differ among populations. We detected weak evidence of population-specific melanin-based skin color within environments after 30 days of rearing and similarly weak differences among populations in the extent of the plastic response in carotenoid-based fin color. Taken together, our results indicate a relatively greater role of the environment rather than genetic control in shaping skin and fin color intensity. More generally, the results presented here support the hypothesis that successful invaders, such as brown trout, display marked morphological phenotypic plasticity and that plasticity, rather than genetic preadaptation could be the initial mechanism facilitating successful colonization.

The melanin-based skin color plasticity we observed in brown trout presumably functions to both match individuals to their surrounding background [12], [22], [56] and to produce disruptive coloration through controlling the contrast of banding marks, called ‘parr marks’, to the rest of the body [24].There has been renewed interest in recent years to better understand the mechanisms responsible for animal camouflage and crypsis along with the associated consequences for fitness [57]. Generally speaking, crypsis can be accomplished by obscuring the outline of an individual to potential predators through background color matching [58] or disruptive coloration patterns [59]. Empirical studies show that color plasticity can influence fitness: coastrange sculpin models painted to more closely match their surrounding substrate were less likely attacked by predators [13], and brook trout fry acclimated to tanks that more closely matched the color of natural streams were less likely to be consumed by avian predators upon release [25]. In addition to the apparent plastic ability to match substrate coloration, brown trout exhibit parr marks that serve to disrupt the outline of the fish when viewed laterally [60]. Though our analysis of skin color included both parr mark and other skin coloration, it appeared that the contrast between the parr marks and the rest of the fish varied between treatments (e.g., see images in Fig. 4). Specifically, the contrast between parr mark and the rest of the fish was greater in the light substrate treatments, suggesting that disruptive coloration could be more important than background matching for predator avoidance [61] in these light-colored environments. The primary sources of predation experienced by these four populations are from birds such as belted kingfishers *Megaceryle alcyon* (Westley, personal observations) and from cannibalism by larger brown trout [46]. It remains unclear how background matching or disruptive color crypsis might protect against avian predators attacking from above, versus piscivores attacking the lateral sides of the trout.

We observed generally consistent patterns between melanin-based skin color and carotenoid-based fin colors. To our knowledge, there is no evidence to suggest that carotenoid-based colors can be self-synthesized by salmonids and researchers continue to assume that these colors must be acquired through the diet [20], [21], [62]. Our results detected marked plasticity in carotenoid-based fin colors, despite all individuals being fed identical carotenoid-rich *Artemia*. One potential explanation might have been that individuals grew more slowly in the dark substrate treatment, acquired less pigment from the *Artemia* and were thus darker-colored. However, this scenario is unlikely as growth did not differ among treatments (ANOVA, p  =  0.2) Our finding of color plasticity in carotenoid-based colors points towards the intriguing possibility of self-synthesis of carotenoid-like colors in salmonids, similar to current findings in guppies [16], [17], [63]. While melanin-based skin colors are clearly used for background matching and disruptive color crypsis, carotenoid-based colors during the juvenile life stage do not have as obvious a function. Carotenoid-based colors are often hypothesized to be involved in sexual displays during reproduction in brown trout, though recent evidence found reproductive success with melanin-based rather than carotenoid-based pigment patterns [21]. In lieu of a reproductive function, carotenoid-based colors might facilitate species recognition between brown trout and similar appearing Atlantic salmon, might be used in territorial displays to conspecifics [31], or serve an immunological or other physiological function [62], [64].

We detected weak evidence of population-level differences in melanin-based skin color within an environment and no evidence of population-level differences in the shape of the plastic response to rearing substrate. Taken together, these findings provide compelling evidence of greater environmental rather than genetic control on the skin coloration intensity we quantified. These findings also countered our predictions that Middle Rocky Brook and Parkers Brook would be more similar to each other than to either the Waterford or Rennies River populations based on habitat similarity [48] and potential patterns of gene flow. This finding suggests that the costs of maintaining plasticity in color intensity are likely to be low [2]. In addition, our results contrast with the recent evidence of heritable color plasticity at the family-level in coastrange sculpins [14]. However, similar to our findings, Morris et al. [65] found that growth reaction norms were parallel among farmed, wild, and hybrid groups of Atlantic salmon suggesting that selection on growth would not alter the shape of the phenotypic response, but perhaps the character state or y-intercepts. We note, however, that earlier work has shown heritable differences in the *number* and *size* of melanin-based [21] and carotenoid-based [66] pigment spots in brown trout. Moreover, parr mark expression appears to have underlying genetic control in closely related Atlantic salmon [38]. Thus, plasticity in color change intensity (as we measured it) might not have a clear heritable basis, but color patterning in the size and shapes of spots or parr marks likely could. Carotenoid color can be transferred from the muscle tissue of mothers to their developing ova [64], [67], indicating a potential role of environmental maternal effects in offspring coloration. While the potential influence of maternal effects is unclear, all families were spawned from comparably similar pink-colored eggs common to lake-rearing or sea-rearing adults.

One of the more difficult aspects of conducting work on phenotypic plasticity results from the fact that different traits, in different environments, can lead to different answers. We tested for evidence of heritable differences in skin color plasticity between two environments that we believed captured the two extreme colors of substrate that these populations would perhaps encounter in their natural settings. That being said, additional environments beyond the two we tested might have induced different plastic responses [2], [26], [68] and thus could have altered our interpretation of the genetic control on color plasticity. Notwithstanding these caveats, it seems plausible that phenotypic plasticity is largely responsible for the differences in the intensity of coloration observed among these populations in nature.

## Supporting Information

Figure S1 **Principal component plot of the 20 top colors derived from image analysis of melanin-based color in brown trout.** Each point represents an individual and the average color of that individual is depicted in RGB space on the plot.
(TIF)

Figure S2 **Principal component plot of the 20 top colors derived from image analysis of carotenoid-based color in brown trout.** Each point represents an individual and the average color of that individual is depicted in RGB space on the plot.
(TIF)
