# Supplementary figures and images for: Experimental Tests for Heritable Morphological Color Plasticity in Non-Native Brown Trout (Salmo trutta) Populations
Source: PLoS One. 2013 Nov 18;8(11):e80401. doi: 10.1371/journal.pone.0080401 (PMC3832361; doi:10.1371/journal.pone.0080401)

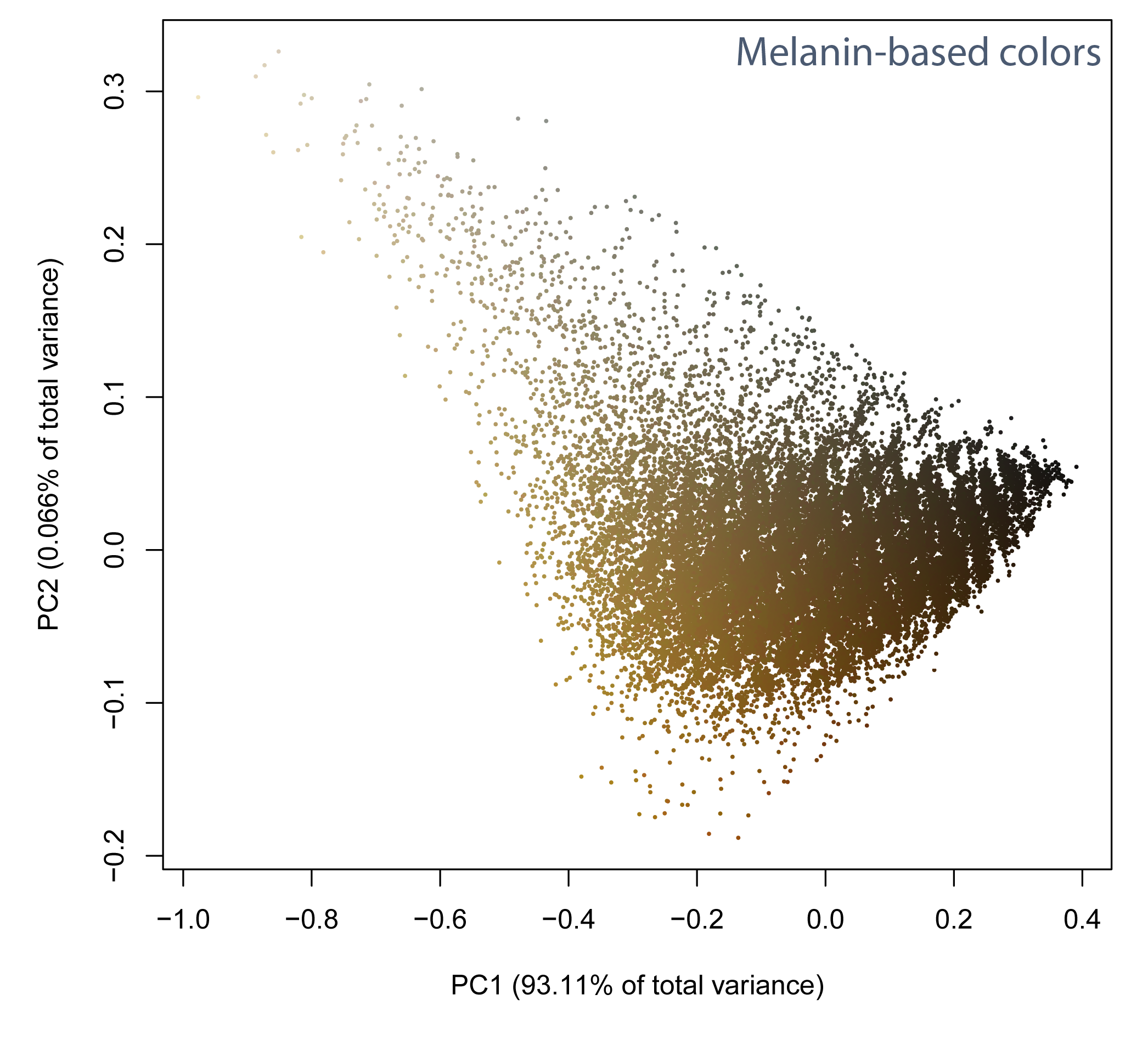

Supplement: Figure S1 — Principal component plot of the 20 top colors derived from image analysis of melanin-based color in brown trout. Each point represents an individual and the average color of that individual is depicted in RGB space on the plot. (TIF) [file pone.0080401.s001.tif]

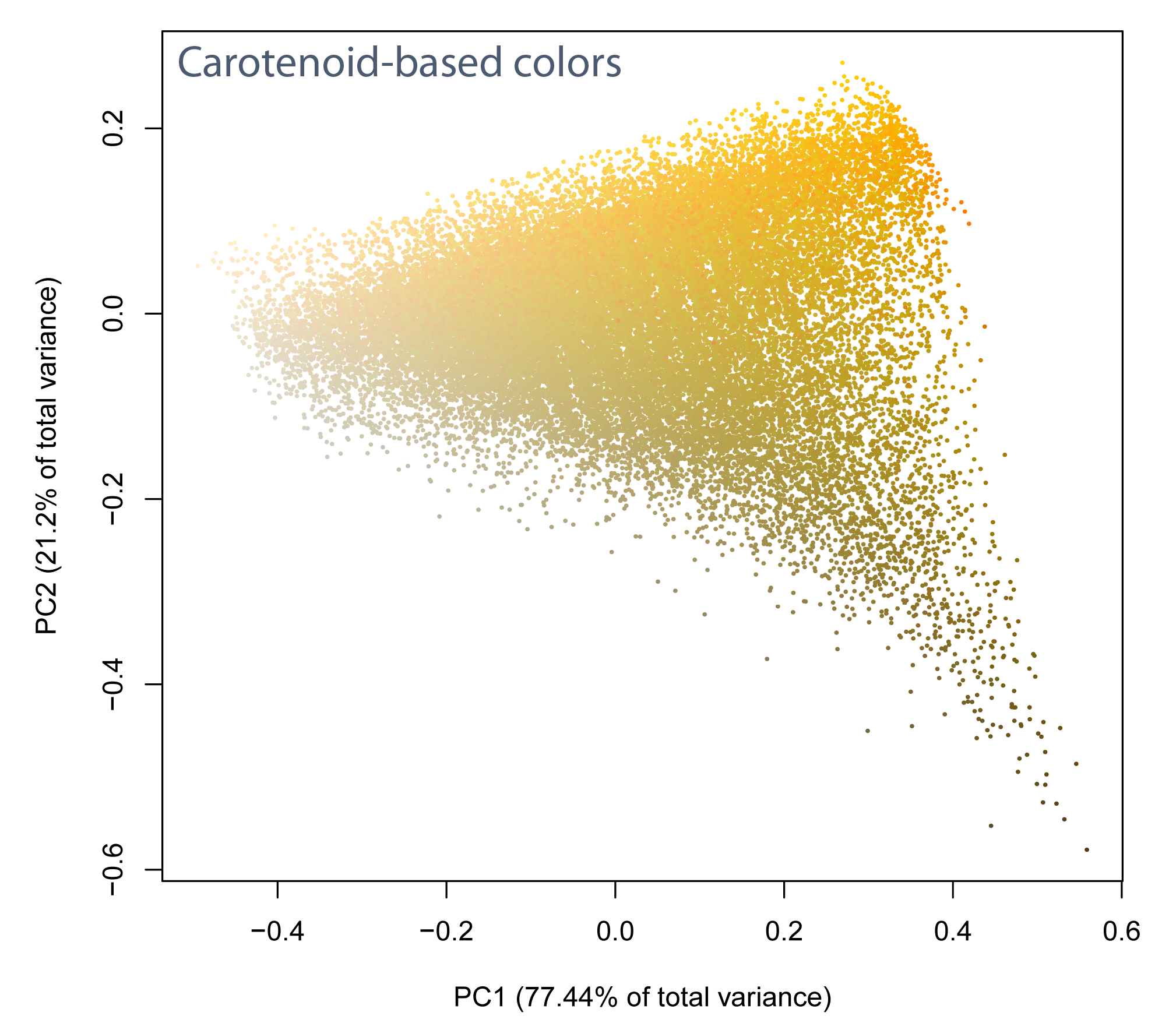

Supplement: Figure S2 — Principal component plot of the 20 top colors derived from image analysis of carotenoid-based color in brown trout. Each point represents an individual and the average color of that individual is depicted in RGB space on the plot. (TIF) [file pone.0080401.s002.tif]
